# Physiological Determinants of PR Interval in Healthy Fetuses: Insights from Correlation and Regression Modeling

**DOI:** 10.3390/jcm14217522

**Published:** 2025-10-23

**Authors:** Grzegorz Swiercz, Katarzyna Janiak, Lukasz Pawlik, Marta Mlodawska, Piotr Kaczmarek, Jakub Mlodawski

**Affiliations:** 1 Intitute of Medical Science, Jan Kochanowski University in Kielce, 25-369 Kielce, Poland; grzegorz.swiercz@ujk.edu.pl (G.S.); marta.mlodawska@ujk.edu.pl (M.M.); 2 Polish Mother’s Memorial Hospital Research Institute, 93-338 Lodz, Poland; kasiajaniak@me.com (K.J.); kaczmarekpiotr1@gmail.com (P.K.); 3 Department of Information Systems, Kielce University of Technology, 25-314 Kielce, Poland; lpawlik@tu.kielce.pl

**Keywords:** fetal PR interval, fetal echocardiography, Doppler ultrasound, multivariable regression

## Abstract

**Background**: The fetal mechanical PR interval (mPR), measured using pulsed-wave Doppler, is a widely used parameter to assess atrioventricular conduction in fetuses, particularly in cases at risk of developing atrioventricular (AV) block. However, the physiological factors that influence mPR readings are not fully understood. This study aimed to identify determinants affecting the measurement of the mPR interval using the mitral valve/aorta (MV/Ao) Doppler method in a cohort of structurally normal fetuses. **Methods**: We retrospectively analyzed 925 fetuses with normal echocardiographic findings and no structural cardiac or extracardiac anomalies. Correlation analysis, group comparisons, trend testing, and multivariable modeling were performed to assess the impact of biometric and Doppler parameters on mPR interval measurements. **Results**: The median mPR interval across the cohort was 116 ms (interquartile range: 108–123 ms). Fetuses were categorized into four gestational age groups (≤19 weeks, 20–23 weeks, 24–27 weeks, and ≥28 weeks). Significant differences in mPR were observed between gestational age groups (*p* < 0.01), with a positive trend across increasing gestational age (*p* < 0.0001). The strongest correlation was an inverse relationship between mPR and fetal heart rate (FHR) (ρ = −0.256, *p* < 0.01). Multivariable regression identified five independent predictors of mPR: lower FHR, greater biparietal diameter (BPD), larger pulmonary valve diameter (PVD), increased fronto-occipital diameter (FOD), and lower umbilical artery pulsatility index (UA PI). The final model explained approximately 9.9% of the variance in mPR interval (R^2^ = 0.099). **Conclusions**: The fetal mPR interval increases with gestational age and is primarily influenced by fetal heart rate, even after adjusting for other factors. Certain biometric and Doppler parameters also contribute modestly to mPR variation. These findings highlight the importance of accounting for physiological variability when interpreting mPR measurements in clinical fetal cardiology.

## 1. Introduction

Evaluation of fetal atrioventricular (AV) conduction is central to assessing pregnancies at risk of maternal autoantibody-mediated complications. This is particularly relevant in women with anti-SS-A/Ro and anti-SS-B/La antibodies, typical of Sjögren’s syndrome but also present in other autoimmune disorders. The mechanical PR interval (mPR) is a surrogate of AV conduction and is used to detect first- and second-degree fetal AV block. Although the PR interval is an electrophysiological measure—on ECG, it spans from the onset of atrial depolarization (P wave) to the start of the QRS complex—several in utero techniques estimate it indirectly.

Approaches include non-invasive fetal ECG (NI-fECG) and fetal magnetocardiography (fMCG), which reconstruct electrical activity from myocardial magnetic fields. Ultrasound-based methods are most widely used in routine care because they are available and practical, including M-mode, tissue Doppler imaging, and pulsed-wave Doppler.

In clinical practice, mPR is usually measured with pulsed-wave Doppler in one of two planes: the five-chamber view sampling mitral inflow and aortic outflow (MV/Ao), or the superior vena cava–aorta plane (SVC/Ao). M-mode has been proposed but can be technically challenging for precise onset timing of atrial and ventricular contraction. Tissue Doppler is less common on standard obstetric systems [1,2].

Objective: To identify and characterize physiological variables associated with variability in fetal mPR measured by the MV/Ao Doppler method.

## 2. Materials and Methods

We included pregnant patients who underwent consultative fetal echocardiography (FE) during the second and early third trimester at the Fetal Cardiology Outpatient Clinic of the Provincial Combined Hospital in Kielce between January 2023 and December 2024. Only fetuses with anatomically and functionally normal echocardiographic findings, and no extracardiac anomalies, were included in the analysis. Exclusion criteria comprised fetuses with confirmed chromosomal abnormalities or a positive non-invasive prenatal testing (NIPT) result, as well as multiple gestations. We also excluded patients who were taking medications that could affect conduction within the cardiac conduction system. Patients referred for FE due to the presence of maternal anti-SS-A (anti-Ro) and anti-SS-B (anti-La) antibodies were also excluded.

The primary outcome was the mechanical atrioventricular conduction time (mechanical PR interval, mPR), measured using pulsed-wave Doppler. The Doppler sample gate was positioned to simultaneously capture inflow through the mitral valve and outflow through the ascending aorta (MV/Ao method), with the fetal heart in a near apex-up or apex-down orientation. The AV conduction time was measured from the onset of the A-wave of mitral inflow to the onset of aortic outflow.

All measurements were performed by a single experienced ultrasonographer—a pediatrician certified in fetal echocardiography with over 20 years of clinical experience. Each measurement was conducted once per patient.

We analyzed the cohort for mPR duration and its association with accompanying physiological parameters.

This was a retrospective observational cohort study approved by the Bioethics Committee at Jan Kochanowski University (approval number: 49/2025). All methods were conducted in accordance with local regulations and the ethical guidelines of the institutional review board. Written informed consent for ultrasound examination was obtained from all participants.

## 3. Statistical Analysis

The study population was stratified by gestational age into four groups: ≤19 weeks, 20–23 weeks, 24–27 weeks, and ≥28 weeks of gestation. For each group, the median, interquartile range (IQR), and first (Q1) and third quartiles (Q3) were calculated for all variables. Due to non-normal distributions, as assessed by the Shapiro–Wilk test, group comparisons were conducted using the Kruskal–Wallis test. Post hoc analyses were performed using Dunn’s test with Bonferroni correction. Trends across ordered groups were evaluated using the Jonckheere–Terpstra test.

Spearman’s rank correlation coefficient (ρ) was used to assess bivariate associations. The strength of correlation was interpreted according to the classification proposed by J. Guilford. Differences between dependent correlations were evaluated using Steiger’s Z-test, based on Fisher’s Z-transformation.

All variables showing statistically significant correlations with mPR were entered into multivariable regression modeling. We applied both ordinary least squares (OLS) linear regression (with variable selection at *p* < 0.05) and least absolute shrinkage and selection operator (LASSO) regression with five-fold cross-validation to minimize predictor redundancy. All variables were standardized using z-scores. Missing values were imputed using the median.

The final model included only those predictors identified as significant by both OLS and LASSO methods. Results are presented as a regression equation and as a ranked list of variable importance, based on standardized LASSO coefficients.

Statistical analyses were performed using Statistica 13.1 (TIBCO Software Inc., Palo Alto, CA, USA) and Python 3.11, with the following libraries: pandas v2.2.2 [3], numpy v1.26.4 [4], scipy v1.12.0 [5], statsmodels v0.14.0 [6], scikit-learn v1.4.0 [7], matplotlib v3.8.3 [8], seaborn v0.13.2 [9], and scikit-posthocs v0.9.0 [10].

## 4. Results

A total of 925 pregnant individuals with normal fetal echocardiographic findings were included in the final analysis. Initially, 1005 cases were assessed, but 8.5% were excluded due to the presence of cardiac or extracardiac abnormalities. The mean maternal age in the study cohort was 31.3 years (range: 16–38; SD = 5.4). The median gestational age was 26 weeks (Q1 = 25, Q3 = 29; IQR = 4 weeks). The distribution of participants across gestational age intervals is presented in Table 1.

The median PR interval across the entire cohort was 116 ms (Q1 = 108 ms, Q3 = 123 ms; IQR = 15 ms). The distribution was non-normal. Median PR interval values were compared between gestational age groups, as shown in Table 2a. The Kruskal–Wallis test indicated significant differences between groups (H = 30.80, *p* < 0.01). Post hoc pairwise comparisons using Dunn’s test with Bonferroni correction are presented in Table 2b.

Significant differences were observed between most gestational age groups, except for adjacent intervals: 20–23 vs. 24–27 and 24–27 vs. ≥28. The most pronounced differences were noted in comparisons involving the ≤19-week group, where the PR interval median was lowest.

**Figure 1:**
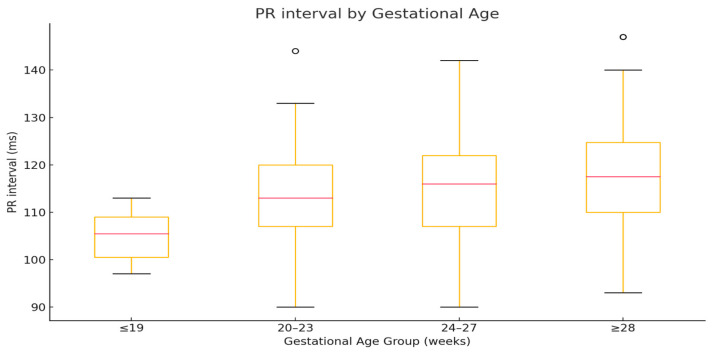
The plot displays group medians (red line), interquartile ranges (boxes), and non-outlier ranges (whiskers), with outliers shown as open circles.

A box-and-whisker plot of PR intervals across gestational age groups is presented in Figure 1. A Jonckheere–Terpstra test confirmed a statistically significant monotonic increasing trend in PR interval with advancing gestational age (J = 143,815; z = −15.69; *p* < 0.0001).

We next analyzed the association between the mPR interval and biometric and Doppler variables. Results of Spearman’s rank correlation are shown in Table 3.

The strongest correlation was observed with FHR (ρ = −0.256), although the strength was weak. mPR was also weakly and positively associated with fetal size parameters, especially those related to head dimensions (BPD, FOD, HC), femur length, and cardiac outflow structures (PVD, AVD). Interestingly, EFW percentile showed weaker association than absolute EFW. Steiger’s Z-test showed no significant difference between the two correlations (ρ = 0.516; z = 1.014; *p* = 0.311).

Among Doppler indices, only UA PI and MCA PSV showed statistically significant associations with mPR, though both were weak.

**Figure 2:**
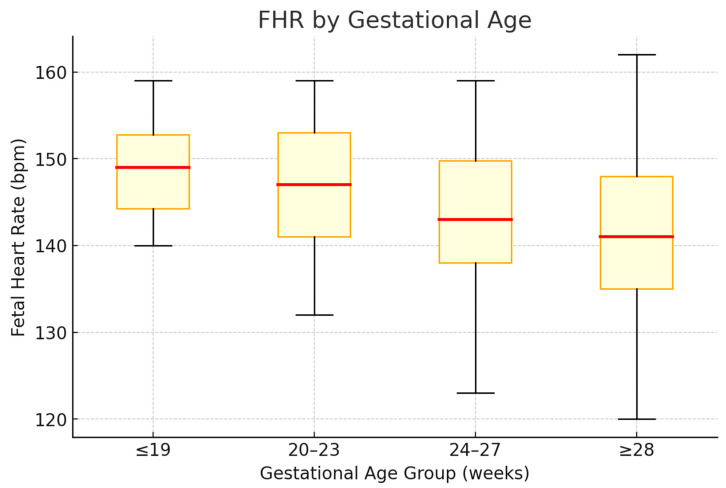
Box-and-whisker plot of FHR across gestational age groups. The red horizontal line denotes the median; the box spans the interquartile range (Q1–Q3); the whiskers extend to the minimum and maximum observed values.

Prior studies have consistently reported an inverse relationship between FHR and mPR [1,11,12], reflecting the physiology of cardiac conduction. In our cohort, FHR also varied significantly across gestational age groups. Figure 2 shows the distribution of FHR medians and quartiles. Table 4 presents group comparisons.

A Jonckheere–Terpstra test demonstrated a significant decreasing trend in FHR across gestational age groups (J = 100,353; z = −26.18; *p* < 0.0001).

Given the correlation between mPR and both FHR and gestational age, we constructed a univariable OLS regression model:

PR interval (ms) = 144.17 + 0.40 × Gestational Age (weeks) − 0.27 × FHR (bpm)(1)

Both coefficients were statistically significant (*p* < 0.001). The model explained 8.3% of the variance in mPR (R^2^ = 0.083), indicating that gestational age remains an independent predictor of PR interval even after adjusting for FHR.

To explore additional predictors, we developed a multivariable model including variables significantly correlated with mPR. Five variables were retained as independent predictors:

PR (ms) = 188.5 − 0.293 × FHR − 1.381 × PVD + 1.746 × BPD + 1.234 × FOD − 3.755 × UA PI(2)

Final model predictors: FHR, UA PI, BPD, FOD, and PVD. These variables were selected by both OLS and LASSO regression. FHR was negatively associated with PR duration. Larger BPD and FOD values, greater PVD, and higher UA PI were associated with longer PR intervals. Gestational age and EFW were excluded due to multicollinearity, as fetal age was represented by biometric dimensions.

The final model explained approximately 9.9% of PR interval variance (R^2^ = 0.099). Predictor importance and standardized coefficients are shown in Table 5.

## 5. Discussion

Fetal PR interval assessment is primarily utilized in the diagnosis and surveillance of autoimmune congenital atrioventricular block (CAVB), which is associated with maternal anti-SS-A and anti-SS-B antibodies—autoantibodies typical for Sjögren’s syndrome but also present in other autoimmune conditions [13]. However, evaluation of AV conduction time may also be relevant in cases of fetal channelopathies, in monitoring disease progression toward complete heart block, in assessing therapeutic effects of maternal treatments (e.g., β-mimetics, corticosteroids, hydroxychloroquine, and intravenous immunoglobulins), and in differentiating types of fetal arrhythmias [14,15].

Fetal PR interval assessment has also been proposed as a potential prognostic marker in evaluating fetal well-being, particularly in the context of acidosis, electrolyte imbalances, or hypoxia. Prior studies have explored its utility in fetal growth restriction (FGR) [16] and intrahepatic cholestasis of pregnancy [17]. In adult populations, both shortened and prolonged PR intervals on ECG are associated with adverse outcomes, such as increased risk of heart failure and need for pacemaker implantation. The relationship is non-linear, with elevated risk observed for PR values < 125 ms and >200 ms [18].

Experimental studies in fetal sheep have demonstrated persistent AV conduction abnormalities following umbilical cord occlusion designed to simulate uterine contractions and induce in utero acidosis. These conduction disturbances did not resolve upon reperfusion and required resuscitation post-delivery [19]. Such findings support the hypothesis that fetal PR interval may serve as a predictive biomarker of stillbirth or adverse intrapartum outcome.

Given the clinical importance of mPR in maternal–fetal medicine, understanding the physiological factors influencing its measurement is essential. Direct fetal ECG acquisition is often technically challenging or infeasible; thus, surrogate markers of cardiac conduction are used, such as the mechanical PR interval assessed by pulsed-wave Doppler. The MV/Ao method has been demonstrated to be reliable, with minimal and statistically insignificant interobserver variability [20].

Early studies in the 2000s suggested that mPR was independent of both fetal heart rate (FHR) and gestational age (GA) [21]. However, Bolnick et al. later reported statistically significant, albeit weaker, correlations between mPR, FHR, and GA—consistent with our findings [22]. In a larger cohort, Wojakowski et al. conducted a quantitative analysis of mPR determinants, establishing normative ranges and exploring the influence of FHR, GA, and fetal sex. Their univariable model indicated an increase in mPR of approximately 0.4 ms per week of gestation and a decrease of 1.4 ms for every 5 bpm increase in FHR. They reported no effect of fetal sex. Their multivariable regression model yielded the equation: mPR (ms) = 143.9 + 0.29 × GA (weeks) − 0.20 × FHR (bpm) [11]—closely resembling our own model with the same two predictors. These authors also noted that correlations between mPR and its determinants remained stable from 16 to 36 weeks of gestation. Like our findings, they observed that FHR and GA independently influence mPR, despite their mutual correlation.

Tomek et al. [23] also demonstrated similar directional correlations between mPR, FHR, and GA. However, in contrast to our study, they did not observe a significant correlation between GA and FHR, which may explain the stronger regression coefficients in their model. Notably, their cohort was considerably smaller, and no sample size estimation was reported, limiting interpretability regarding potential type II error in the FHR–GA relationship.

Some authors have suggested that the association between FHR and mPR may be secondary, with lower heart rates seen in more advanced gestation [12]. However, our multivariable regression model suggests the opposite: that FHR exerts a stronger independent effect on mPR than GA. In a Polish cohort, the correlation between gestational age and mPR was 0.22, while the correlation between mPR and FHR was −0.39 [1]. That study did not find significant differences in mPR between GA-stratified groups based on differing FHR ranges. However, those comparisons were limited by small sample sizes, increasing susceptibility to type II error.

In addition, some researchers have demonstrated that PR interval measurement by pulsed Doppler may also be feasible postnatally. Bergman et al. [24] reported a strong correlation between mPR obtained by the MV–Ao method and PR interval measured by ECG (r = 0.82).

Pulsed Doppler assessment of mPR is not the only technique available for evaluating fetal AV conduction. Non-invasive electrophysiological assessment (NIEA) includes fetal electrocardiography (NIFECG)—which records cardiac electrical activity via abdominal electrodes—and fetal magnetocardiography (fMCG), which captures magnetic field changes induced by propagation of electrical impulses through the conduction system. (16)

Mechanical PR interval measured by pulsed Doppler correlates with the electrical PR interval obtained by fMCG. However, mPR values tend to be, on average, 14.6% higher (95% limits of agreement: −8.6 to +38.0 ms using the SVC/aAo method), which may lead to overestimation and potential overdiagnosis of first-degree AV block [12]. Another study reported a mean positive bias of 16.47 ms (95% CI: −43.43 to +10.44 ms), with slightly lower inter- and intraobserver reliability compared to fMCG—though correlations remained strong (Pearson’s r > 0.9) [25]

Some studies have demonstrated statistically significant shortening of PR intervals (measured by fMCG) in fetuses with FGR or maternal hypertension [26]. This may be related to sympathetic activation (e.g., alpha-adrenergic tone) in hypoxic fetuses, which exerts a positive chronotropic effect and promotes blood flow redistribution. However, not all studies have confirmed the discriminatory value of PR interval in distinguishing FGR from appropriately growing fetuses [27].

fMCG provides greater precision by evaluating the true electrical PR interval (via PQ segment) with higher temporal resolution and reduced sensitivity to fetal position, movement, or blood flow turbulence [28]. Despite its advantages, fMCG has not been widely adopted into clinical practice due to its high cost and limited availability. Pulsed Doppler mPR remains the most widely used method for PR interval assessment in routine fetal surveillance due to the accessibility of ultrasound and ease of acquisition.

In our study, we found a very weak but statistically significant correlation with the umbilical artery pulsatility index (UA PI), and the direction of the association was negative. This relationship may have a pathophysiological basis: hypoxemia—which is plausibly linked to higher UA PI—can increase vagal tone and thereby slow the fetal heart rate. Our study also shows a weak positive correlation between the mechanical PR interval (mPR) and estimated fetal weight (EFW), while independent studies indicate that EFW is associated with UA PI [29].

Our study aimed to characterize physiological determinants of the fetal mPR interval. We identified variables associated with mPR and defined the direction of their effects. Notably, in our multivariable model ~90% of the variance in mPR remained unexplained by the accompanying variables, indicating a high degree of independence. While several potential covariates were not evaluated in this study, the evidence strongly suggests that mPR reflects the function of the cardiac conduction system, whose autonomy is also apparent in our regression analysis. This supports the diagnostic reliability of mPR in clinical settings with suboptimal measurement conditions, particularly in high-risk pregnancies undergoing evaluation for atrioventricular block.

Given the serious consequences of complete AV block in the neonate and the limited safety data on current preventive strategies—due to the rarity of the condition—fetal PR interval prolongation must be interpreted with confidence and reproducibility.

Limitations. This study did not include comparison with reference techniques such as fetal magnetocardiography (fMCG) or non-invasive fetal electrocardiography (NI-fECG). Nevertheless, we used the most commonly applied method for PR-interval assessment, whereas these alternative techniques are largely confined to research settings in specialized centers because of their cost and technical demands. Our data reflect a real-world cohort; therefore, potential confounders—such as fetal movement and maternal body habitus—were not systematically controlled. The study was conducted in a tertiary referral center; although fetuses with structural anomalies were excluded, the cohort should be considered high-risk and susceptible to selection bias. The retrospective design also increases the risk of missing data. Finally, because the study population was drawn from a high-risk referral group undergoing serial fetal echocardiography—and only fetuses with structurally and functionally normal hearts were included—generalizability to the broader obstetric population may be limited.

## 6. Conclusions

Fetal mechanical PR interval increases with advancing gestational age, and this trend is partially independent of fetal heart rate (FHR). Nevertheless, FHR remains the strongest individual predictor of mPR. In addition to FHR, mPR is influenced by umbilical artery pulsatility index (UA PI) and larger fetal biometric dimensions.

Pulsed-wave Doppler measurement of mPR using the MV/Ao method is a practical and clinically applicable technique; however, it requires cautious interpretation due to its sensitivity to FHR and select biometric parameters.

## Figures and Tables

**Table 1:** Distribution of study participants by gestational age.

Gestational Age (Weeks)	Count	% of Total	Cumulative %
16–19	19	2.054054	2.054054
20–23	101	10.91892	12.97297
24–27	461	49.83784	62.81081
28+	344	37.18919	100

**Table 2:** (**a**) Median PR interval by gestational age group. (**b**) Post hoc pairwise comparisons (Dunn’s test, Bonferroni correction). ns—not significant.

**(a)**				
**Group**	**Median**	**Q1**	**Q3**	**IQR**
≤19	105.5	100.5	109	8.5
20–23	113	107	120	13
24–27	116	107	122	15
≥28	117.5	110	124.75	14.75
**(b)**				
**Comparison**		**Significance**		
≤19 vs. 20–23		*p* < 0.01		
≤19 vs. 24–27		*p* < 0.01		
≤19 vs. ≥28		*p* < 0.01		
20–23 vs. 24–27		ns		
20–23 vs. ≥28		*p* < 0.05		
24–27 vs. ≥28		ns		

**Table 3:** Correlation between mPR interval and biometric/Doppler variables. ns—not significant.

Variable	Spearman’s ρ	*p*-Value
Fetal Heart Rate (FHR)	−0.256	<0.01
Femur Length (FL)	0.174	<0.01
Pulmonary Valve Diameter (PVD)	0.169	<0.01
Biparietal Diameter (BPD)	0.168	<0.01
Head Circumference (HC)	0.168	<0.01
Fronto-Occipital Diameter (FOD)	0.167	<0.01
Abdominal Circumference (AC)	0.163	<0.01
Gestational Age	0.159	<0.01
Estimated Fetal Weight (EFW)	0.159	<0.01
Aortic Valve Diameter (AVD)	0.155	<0.01
Femur Length (FL)	0.151	<0.01
Middle Cerebral Artery Peak Systolic Velocity (MCA PSV)	0.103	<0.05
Umbilical Artery Pulsatility Index (UA PI)	−0.084	<0.05
Estimated Weight (Percentile)	0.071	<0.05
Aortic-to-Pulmonary Valve Ratio (Ao/TP)	−0.07	<0.05
Head-to-Abdominal Circumference Ratio (HC/AC)	−0.093	ns
Pulsatility Index of Ductus Venosus (PIV)	−0.065	ns
Cerebroplacental Ratio (CPR)	0.052	ns
Middle Cerebral Artery Pulsatility Index (MCA)	0.038	ns
Biparietal-to-Fronto-Occipital Ratio (BPD/FOD)	0.032	ns
Biparietal-to-Femur Length Ratio (BPD/FL)	−0.032	ns

**Table 4:** (**a**) Comparison of median fetal heart rate (FHR) across the four gestational age groups revealed statistically significant differences between groups (Kruskal–Wallis test: H = 41.21, *p* < 0.01). (**b**) Post hoc pairwise comparisons using Dunn’s test with Bonferroni correction identified significant differences (*p* < 0.05) between selected group pairs with respect to median values. ns—not significant.

**(a)**				
**Group**	**Median**	**Q1**	**Q3**	**IQR**
≤19	149	144.25	152.75	8.5
20–23	147	141	153	12
24–27	143	138	149.75	11.75
≥28	141	135	148	13
**(b)**				
**Comparison**		**z**	***p***	
≤19 vs. 20–23		1.118602	ns	
≤19 vs. 24–27		2.603423	ns	
≤19 vs. ≥28		3.692449	*p* < 0.01	
20–23 vs. 24–27		3.087323	*p* < 0.05	
20–23 vs. ≥28		5.356437	*p* < 0.01	
24–27 vs. ≥28		3.738765	*p* < 0.01	

**Table 5:** Standardized regression coefficients (β) from LASSO model.

Variable	Standardized Coefficient	Direction
Fetal Heart Rate (FHR)	−2.011	Negative
Biparietal Diameter (BPD)	0.644	Positive
Pulmonary Valve Diameter (PVD)	0.392	Positive
Umbilical Artery Pulsatility Index (UA PI)	−0.382	Negative
Fronto-Occipital Diameter (FOD)	0.033	Positive
Model R^2^	0.099	Explains ~9.9% of PR interval variability

## Data Availability

The dataset supporting this study has been deposited in a public repository and is accessible at the following URL: https://doi.org/10.17605/OSF.IO/E3XSH (accessed on 1 August 2025).
